# Low Rates of Hardware Removal and Tendon Rupture for the Acu-Loc 2 Volar Distal Radius Plate: A Minimum One-Year Follow-Up Study

**DOI:** 10.7759/cureus.62165

**Published:** 2024-06-11

**Authors:** Aniket Bharadwaj, Nimalesh Yogarajah, Warran Wignadasan, Anthea Davy, Alistair R Hunter

**Affiliations:** 1 Trauma and Orthopaedics, University College London Hospital, London, GBR; 2 Trauma and Orthopaedics, Royal Free Hospital NHS Trust, London, GBR

**Keywords:** wrist trauma, distal locking plate, distal end radius plating, distal end radius fracture, volar locking plate

## Abstract

Aim: Volar locking plates designed for far distal radius fracture fixation can have a significant hardware removal rate and risk of tendon rupture. Plate design has a role in the rate of complications. This study assessed the hardware removal and tendon rupture rate of the Acu-Loc 2 volar distal radius (VDR) plate often used in the treatment of far distal radial fractures.

Method: We searched our electronic healthcare records system for all patients who had undergone fixation with an Acu-Loc 2 VDR plate (Acumed, Hillsboro, OR, USA) at a tertiary center between January 2017 and December 2021. Patients were excluded if their follow-up time was less than one year or if they could not be contacted by telephone follow-up. Pre-operative radiographs were examined for fracture classification. Follow-up time was defined as the last contact in the clinic or by telephone.

Results: A total of 92 patients underwent an open reduction and internal fixation (ORIF) with an Acu-Loc 2 VDR plate. A total of 85 patients met the inclusion criteria for this study. Our cohort included 33 males (38.8%) and 52 females (61.2%). The mean age was 50 years. Twenty-seven fractures (31.0%) were extra-articular, and 60 fractures (69.0%) were intra-articular. The mean follow-up time for the patients was 593.3 days (range 369 to 1185 days). Four patients (4.7%) had their hardware removed. Three (3.5%) patients underwent removal due to tendon irritation and one patient (1.2%) due to a peri-prosthetic fracture around the plate. There were no tendon ruptures recorded.

Conclusion: The Acu-Loc 2 VDR plate had a low medium-term hardware removal rate and no tendon ruptures. These rates are lower than would be expected when compared with other far distal plate designs.

## Introduction

Volar locking plates (VLPs) are widely used to treat distal radius fracture [1-3]. Complications, such as tendon rupture or metalwork removal for soft tissue irritation or joint penetration are a cause for concern [4,5]. Rates of VLP removal vary from 0 to 100%, in many cases dependent on the center or country [6-12]. The design of the VLP can determine the likelihood of these complications, with those more prominent at the volar rim or placed in a more distal position more likely to cause soft tissue irritation [13,14].

Far distal radius fractures can be challenging to treat. A number of operative methods have been employed to tackle the difficulties posed by far-distal fractures and these include bridging plate techniques, spanning external fixators, fragment-specific plates, or fine wire techniques for specific fragments [15-17]. More recent anatomical VLP designs can be categorized into those placed proximal to the watershed line and those applied to this site. The use of these more proximal VLPs for far-distal radius fractures will offer inadequate fixation and stability or often require additional instrumentation to ensure the volar rim is confidently addressed. Moreover, attempting to capture far-distal fragments using a standard VLP increases the risk of radiocarpal joint penetration. VLPs that sit on the watershed line allow surgeons to address these more difficult fractures. Despite developments in plate design over the past two decades, the original articles reporting high rates of metalwork removal and tendon ruptures are still quoted [18,19]. Our institute has used Acu-Loc 2 (Acumed, Oregon, USA) VLPs to fix distal radius fractures since 2017. The Acu-Loc 2 VDR plate is specifically designed to allow far-distal fractures to be stabilized. The distal row of screws is placed perpendicular to the distal aspect of the plate, allowing for the plate to be placed more distally on the radius. The plate is very low profile distally, theoretically reducing the risk of flexor tendon rupture or soft tissue irritation when compared to other far distal plate designs. Some far distal VLP designs require routine removal once the fracture has healed, due to concerns about the prominence of the plate at the watershed region [7]. We assessed the metalwork removal and tendon rupture rate for the Acu-Loc 2 VDR plate in our patient cohort.

## Materials and methods

We conducted a retrospective review of the case notes and radiographs from our electronic patient record system (Epic Systems Corporation, Wisconsin, USA). Consecutive patients undergoing fixation of their distal radius fracture with an Acu-Loc 2 VDR plate between January 2017 and December 2021 were included. Radiographs were reviewed by three of the authors (AB, NY, and WW). Demographic data that was collected included sex, age, side of the operation, fracture classification (intra- vs extra-articular fractures), total follow-up time (calculated as the time from operative fixation to the date of last contact in the clinic or by telephone by either a clinician or a member of our hand therapy team) and whether there was any documentation of hardware removal at our institution. Patients were contacted retrospectively as part of routine follow-up, and following verbal consent, asked whether their VLP was still in situ or removed postoperatively. Moreover, patients were given the opportunity to explain any symptoms they had been experiencing since discharge. Any symptoms such as irritation or ongoing pain were ascertained.

**Figure 1:**
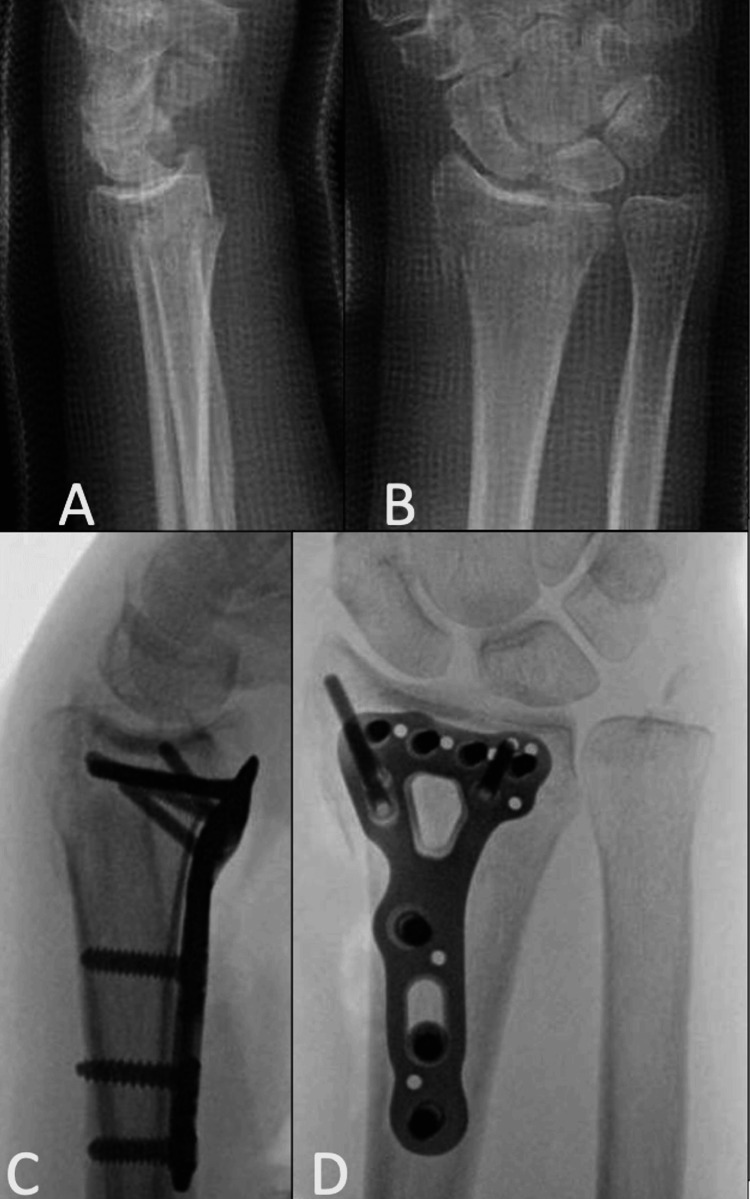
Pre-operative (A-B) and fluoroscopic intra-operative images (C-D) of a female patient aged 58 demonstrating fixation with an Acumed Acu-Loc 2 on the distal radius. The fracture shown is a left-sided intra-articular distal radius fracture, fixed via open reduction and internal fixation (ORIF) using the Acumed Acu-Loc 2 VDR plate. (C-D) The images show far distal placement that is attributed to this plate type.

Patients that were excluded from the study were those who had less than one year of follow-up and those whom we were unable to contact. None of the patients that we successfully contacted wanted to opt out of being included in the study. We recorded which patients had removed their hardware and noted the reason for hardware removal. Figure 1 depicts pre-operative radiographs of a patient who underwent operative fixation using an Acu-Loc 2 VDR plate as well as fluoroscopic images with the plate in situ.

## Results

A total of 92 underwent distal radius fixation using an Acu-Loc 2 VDR plate between January 2017 and December 2021. Of these patients, 85 met the inclusion criteria. Our patient cohort consisted of 33 males (38.8%) and 52 females (61.2%), with an age range of 18-81 years. Radiographs were analyzed to assess for the type of fracture extension, of which 60 cases (70.6%) were intra-articular and 25 cases (29.4%) were extra-articular fractures.

**Figure 2:**
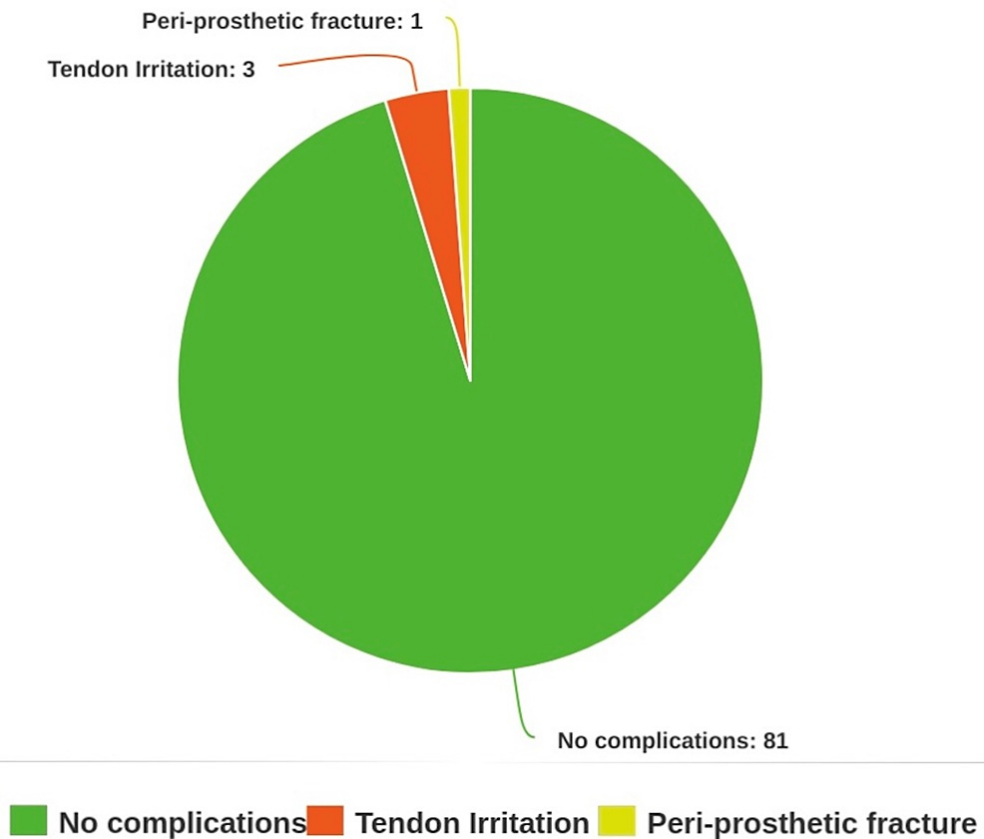
Breakdown of patients followed up in the study, including reasons for plate removal where applicable. The figure is drawn by the authors of this article.

The mean follow-up time was 593.3 days (range 369 to 1185 days). A total of four (4.7%) patients underwent removal of their metalwork. Three patients (3.5%) had their metalwork removed as a result of tendon irritation (86, 366, and 377 days after the initial fixation). Two of these three patients had their plates removed at our institution and one at another institution closer to their residence. These three patients all suffered from pain or irritation when extending their wrists. None of these patients experienced any attritional tendon ruptures as a result of the VDR. One patient (1.2%) sustained a periprosthetic fracture after a further fall, which required the removal of the Acu-Loc 2 plate and re-fixation with the use of a longer extra-articular plate. This occurred 551 days postoperatively and the re-fixation occurred at our institution. This particular patient did not have any complications from the metalwork prior to their second fall and was clinically and functionally doing well and had been discharged from our services prior to fracture. The complications are illustrated in Figure 2.

## Discussion

Our metalwork removal rate for the Acu-Loc 2 VDR plate was 4.7%, with a minimum follow-up of one year. This is significantly lower than a previous study showing a 21% removal rate for a patient cohort where distal radius fractures were repaired using Acu-Loc 1 and VariAx Strkyer VLPs [20]. The design of some previous plates led to a higher revision rate in part due to incomplete restoration of the anatomy of the radius (fragmented pieces were either shortened or incorrectly angled) [20]. The Acu-Loc 2 plate is contoured to the volar aspect of the distal radius and is low profile distally, allowing fracture segments to be adequately captured. The causes of plate removal in our cohort match the complications associated with VLP insertion reported in other series. Studies have shown that flexor tenosynovitis is the most common complication following VLP insertion [7,21,22].

Brown et al. conducted a retrospective study involving 798 patients who underwent fixation with either the Acu-Loc 2 VDR plate, the Acu-Loc 2 proximal VDR plate, or the DVR plate for their distal radius fractures [23]. The study aimed to assess the rate of tendon rupture using the same methodology as the original articles which quote high tendon rupture rates after use of a VLP. They found a tendon rupture rate of just 0.5% and a metalwork removal rate of 10%. No patients in our cohort sustained tendon rupture at minimum one-year follow-up and our metalwork removal rate was lower.

Minegishi et al. 2011 retrospectively reviewed Acu-Loc plate complications and reported no flexor tenosynovitis, infection, or non-union. However, the flexor pollicis longus tendon rupture rate was 6.7% [21]. Another study published in 2011 revealed a rupture rate of 4% with the use of Acu-Loc plates on 73 distal radius fracture fixations [13]. This could be explained by the placement of the Acu-Loc plates at the watershed line of the distal radius, where the least distance between the bone and the flexor tendons exists, leading to a greater risk of friction-related tendon attrition. The first-generation Acu-Loc plates were not as low profile distally as the second-generation Acu-Loc 2 VDR plates. The lack of tendon ruptures in our cohort would indicate the low-profile design may have an advantage. One case of periprosthetic fracture occurred in our sample. There is some evidence that periprosthetic fractures around the screw line may increase with the use of the stiff second-generation hexalobe screws which can be used in the fixation of Acu-Loc plates [24]. In our center, the second-generation hexalobe screws are not routinely used.

There are some limitations to our study. This is a single-center study in an urban teaching hospital. The patient demographic may be skewed toward a younger population. Due to the study being retrospective, some patients were lost to follow-up. Patients were followed up between 12 and 39 months after initial fixation. There is some evidence that flexor tendon rupture could occur up to 68 months after initial surgery [22]. Patients who have not reported any issues or indications for plate revision may develop complications later. Our data showed that 75% of plate removals occurred as a result of tendon irritation and pain. Since pain is a subjective measure, it could be likely that some of the patients may have a greater pain tolerance and, hence, have not opted to present for revision.

## Conclusions

Acu-Loc 2 VDR plates are placed more distally compared to standard VLPs, allowing fixation of far distal radius fractures. Our study describes a low metalwork removal rate and no tendon ruptures with a minimum one-year follow-up. There is no need for routine removal of metalwork, unlike some far distal plate designs. A larger multi-center cohort, coupled with longer follow-up periods, would be beneficial in further giving insight into these findings.
